# Comparison of Two Lateral Flow Immunochromatographic Assays for Rapid Detection of KPC, NDM, IMP, VIM and OXA-48 Carbapenemases in Gram-Negatives

**DOI:** 10.3390/microorganisms13092140

**Published:** 2025-09-12

**Authors:** Clara Morales-Dominguez, Saoussen Oueslati, Nahed Al Laham, Réva Nermont, Hervé Volland, Thierry Naas

**Affiliations:** 1 Maimónides Biomedical Research Institute of Córdoba, 14004 Córdoba, Spain; clara.morales.sspa@juntadeandalucia.es; 2 Reina Sofía University Hospital, 14004 Córdoba, Spain; 3 CIBER de Enfermedades Infecciosas (CIBERINFEC), Instituto de Salud Carlos III (ISCIII), 28029 Madrid, Spain; 4 Team ‘Resist’ UMR1184 Immunology of Viral, Auto-Immune, Hematological and Bacterial Diseases (IMVA-HB), INSERM, Faculty of Medicine, Université Paris-Saclay, CEA, IO Healthi, 94270 Le Kremlin-Bicêtre, France; 5 Bacteriology-Hygiene Unit, Bicêtre Hospital, Assistance Publique-Hôpitaux de Paris, 94270 Le Kremlin-Bicêtre, France; 6 Department of Laboratory Medicine, Faculty of Applied Medical Sciences, Al Azhar University, Gaza P600, Palestine; 7 Médicaments et Technologies Pour la Santé (MTS), Université Paris-Saclay, CEA, INRAE, SPI, 91191 Gif-sur-Yvette, France; herve.volland@cea.fr; 8 French National Reference Center for Antimicrobial Resistance, 94270 Le Kremlin-Bicêtre, France

**Keywords:** carbapenem-resistant Enterobacterales, ceftazidime–avibactam, multidrug-resistant gram-negative bacteria, LFIA

## Abstract

The spread of carbapenemase-producing Gram-negative bacteria poses a significant clinical challenge due to their association with severe Difficult-to-Treat nosocomial infections, as available therapies are drastically reduced. Rapid and accurate detection of carbapenemase-producing Gram-negative bacteria is critical for effective patient management, guiding appropriate antibiotic therapy, and implementing infection control measures to limit their dissemination within healthcare settings. Lateral flow immunoassays that detect the five main carbapenemases have become cornerstones in the fight against carbapenemase-producing Gram-negative bacteria. Carbapenemases evolve in response to antibiotic exposure, and therefore regular evaluation of these lateral flow immunoassays is crucial. Here, we have evaluated a novel assay, the KINVO assay (Medomics Medical Technology) and compared it to the Gold Standard of LFIAs for carbapenemase detection, the NG-TEST CARBA 5 assay (NG-Biotech) on a large panel of carbapenemase variants. The comparison between the two assays highlighted that both share key advantages such as rapidity and simplicity. However, NG-Test CARBA 5 demonstrated superior performance overall, particularly in accurately detecting IMP-type carbapenemases and the OXA-48 variant OXA-505. In contrast, the KINVO assay was more effective at detecting a broader range of KPC variants, including some that have lost carbapenem-hydrolyzing activity but gained resistance to ceftazidime/avibactam. If we consider these variants no longer as carbapenemases, and thus that they should not be detected, the NG-Test CARBA 5 performed better for KPC carbapenemase detection.

## 1. Introduction

Infections with multidrug-resistant (MDR) Gram-negative bacteria (GNB) are increasingly described worldwide and associated with significant morbidity and mortality, thus posing a serious threat to public health [1]. Rapid detection of pathogenic bacteria is essential for timely infection control, appropriate antibiotic stewardship, and improved patient outcomes [1,2]. However, identifying antibiotic resistance remains a challenging and time-consuming process, often taking more than 24 to 48 h and incurring significant costs, particularly with advanced techniques like multiplex PCR assays [3]. Developing faster, cost-effective diagnostic methods is critical to enhance clinical decision-making and reduce the spread of resistant bacteria.

The most widespread carbapenemases in Enterobacterales are classified into three main groups based on their Ambler classification: (i) Class A (Serine β-lactamases), predominantly represented by KPC (Klebsiella pneumoniae carbapenemase) enzymes; (ii) Class B (Metallo-β-lactamases, MBLs), including enzymes such as NDM (New Delhi metallo-β-lactamase), VIM (Verona integron-encoded metallo-β-lactamase), and IMP (Imipenemase); (iii) Class D (Oxacillin-hydrolyzing β-lactamases), mainly represented by OXA-48-like enzymes [4]. Lateral flow immunoassays (LFIAs) have become a key tool for AMR detection, especially for the detection of the five main carbapenemases in cultured GNBs, demonstrating significant advantages, including ease of use without specialized training, rapid turnaround times of less than 15 min, and high reliability, with nearly 100% sensitivity and specificity [5,6,7]. Additionally, LFIAs have been effectively applied directly to clinical specimens such as positive blood cultures, urine samples, and rectal swabs, enabling prompt identification of carbapenemase producers in various clinical settings [8,9,10].

The NG-Test CARBA 5 assay (NG-Biotech, Guipry-Messac, France), that detects the ‘big five’ carbapenemases (KPC, OXA-48-like, NDM, VIM and IMP), has been evaluated worldwide, and its test performances have recently been summarized in a meta-analysis that revealed a sensitivity and specificity, as compared to PCR, of 0.97 [95% CI (0.97, 0.98)] and 0.99 [95% CI (0.99, 1.00)] [7]. NG-Test CARBA 5 has demonstrated superior performance compared to other competitor assays such as RESIST-5 K.O.N.V.I (Coris-BioConcept, Gembloux, Belgium), CP-5 (ERA-Bio, Pittsford, NY, USA), and CRE-LFA (Dynamiker Bio-technology, Tianjin, China). When evaluated using a well-characterized collection of Enterobacterales isolates, NG-Test CARBA 5 exhibited particularly notable accuracy in detecting IMP variants. This is especially significant because IMP carbapenemases are among the most challenging to identify due to their high variability, which complicates detection efforts. Overall, NG-Test CARBA 5’s enhanced sensitivity and specificity in identifying these difficult variants highlight its robustness and reliability in clinical diagnostics [11,12,13].

**Figure 1:**
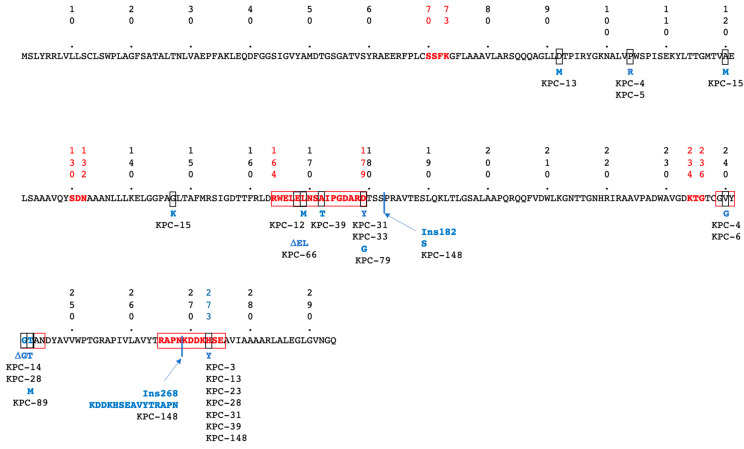
Amino acid (AA) sequence of KPC-2 according to Ambler numbering scheme [20]. Conserved Class A ß-lactamase canonical boxes are indicated in bolded red letters. Regions of AA changes associated with CAZ/AVI resistance are indicated by red boxes. Changed AAs, are highlighted by black boxes; the corresponding AA changes are indicated in blue, along with the KPC variant number in which they have been found. Sites of AA insertions are indicated by black lines, and deleted AAs are indicated by the Greek symbol Δ.

Concurrent with the spread of these carbapenemases, novel variants of each enzyme are increasingly described. As of July 2025, more than 68, 264, 84, 95, and 111 variants of OXA-48, KPC, NDM, VIM, and IMP have been described, respectively (http://www.bldb.eu, accessed on 25 July 2025 [14]). While all MBL variants hydrolyse carbapenems, for OXA-48 and KPC the situation is different. Indeed, 16 OXA-48 variants with 2–4 AA deletions in the ß5–ß6 loop have lost carbapenem-hydrolytic activity but gained expanded-spectrum cephalosporin hydrolysis, thus becoming OXA-ESBL [15]. This is best evidenced with OXA-163 and OXA-405 [16]. The isolation of these variants remains rare, except in Argentina, where OXA-163 is identified in 17%, either alone (6%) or in combination with KPC or NDM (11%), of CPE cases [17]. With the clinical use of a ceftazidim/avibactam (CAZ-AVI) combination to treat infections with KPC-producing Enterobacterales, resistance to this last-resort antibiotic appeared rapidly [18]. Point mutations, insertions, or deletions that led to CAZ-AVI resistance are located within three regions of KPC carbapenemase: the Ω loop (163–179; 134 variants), the S3 strand next to the conserved KTG motif (237–245; 23 variants), the 270-loop (266–275; 34 variants), and in the Ω loop and 270-loop (36 variants) (Figure 1). Changes in the Ω loop at position D179 to Y, and to lesser extent to/N/G/A/V/E/S, correspond to 46 variants (Figure 1). The substitutions in positions 164–179 of the KPC Ω loop are critical for modulating enzymes’ substrate specificity and inhibitor susceptibility. These mutations can enhance the affinity of KPC enzymes for ceftazidime, a third-generation cephalosporin, thereby increasing their ability to hydrolyze this antibiotic and reduce their binding affinity for avibactam. Importantly, these mutations result in loss of carbapenem-hydrolyzing activity but a gain of enhanced activity against ceftazidime; thus, these enzymes effectively transition from carbapenemases to extended-spectrum β-lactamases (ESBLs). This shift in enzymatic profile impacts the pattern of antibiotic resistance and can influence treatment strategies [18,19].

Recently, a novel LFIA KPC/IMP/NDM/VIM/OXA-48 Combo Test Kit (KINVO, Medomics Medical Technology, Changzhou, China), was compared to NG-Test CARBA 5 using 38 CPEs, including a large proportion (25%) of CAZ/AVI-resistant and carbapenem-susceptible KPC variants (including KPC-31 and KPC-33, with D179Y mutations in the Omega loop) [21]. As these variants are not detected by NG-Test CARBA 5, the test performances of the latter were lower than those of the KINVO assay, that detects them [21].

Here, we have extended the comparison of the KINVO assay with the NG-TEST CARBA 5 assay by including a larger panel of carbapenemase variants (114 vs. 38) than used in previous comparisons [12]. In addition, the KPC variants tested have been evaluated using a home-made Carba NP test and PCR.

## 2. Materials and Methods

### 2.1. Bacterial Isolates

The study evaluated 114 well-characterized GNB isolates exhibiting reduced susceptibility to carbapenems. Among these, 109 were carbapenemase-producing (CP) GNBs, including eight VIM-, 24 OXA-48-, 21 KPC-, 15 NDM-, 19 IMP-, 13 multiple carbapenemase-, and nine rare carbapenemase-producers (Table 1). In addition, 5 isolates were classified as non-carbapenemase-producing Enterobacterales (non-CPEs). All bacterial isolates were cultured on Mueller–Hinton agar (Bio-Rad, Marnes-la-Coquette, France) for subsequent testing.

### 2.2. Lateral Flow Immunoassays

For NG-Test CARBA 5, three single colonies were lightly touched with a plastic spreader and then resuspended in five drops of lysis buffer by vortexing. Subsequently, 100 μL of the suspension were directly added to the sampling hole of the test cassette, following the manufacturer’s instructions (NG-Biotech, Guipry-Messac, France, Figure S1A).

For KINVO, three 1µL inoculating loops full of bacteria were resuspended into prefilled tubes containing the extraction buffer and vortexed for 10 s, as recommended by the manufacturer (Medomics Medical Technology, Hangzhou, China). Then, four drops were added to the sampling hole of the test cassette (Figure S1A).

The time to positivity of the test bands and their intensity after the 15 min migration were recorded using a gold color card scale (NG Biotech, Supplementary Figure S1D) [12]. Both assays were performed on the same day from the same bacterial culture, and the results were visually assessed after 15 min by two independent observers who were blinded to the identity of the tested isolates.

### 2.3. Susceptibility Testing, and Carbapenemase Detection

MIC values for selected antibiotics of KPC-producers were determined using E-tests (BioMérieux, Marcy-l’Etoile, France) and interpreted using EUCAST 2025 (https://www.eucast.org/ast_of_bacteria, accessed on 25 July 2025) guidelines (Table 2). Carbapenemase activity was assessed using the home-made Carba NP test as previously described [22]. The PCR-based technique used specific primers (5′ CTGTCTTGTCTCTCATGGCC3′; 5′ CCTCGCTGTGCTTGTCATCC 3′) [23].

### 2.4. Statistical Analysis

The data were collected and managed with Microsoft Excel 2024. Statistical analyses were performed with R software version 4.3.0. The level of significance for statistical tests was set at *p* < 0.05.

## 3. Results

### 3.1. LFIA Testing

The results indicate that both assays demonstrated perfect specificity (100%) in detecting the five targeted enzymes, with confidence intervals of 73.24% to 100%. This means no false-positive results were observed across the assays. Additionally, the sensitivities were high, at 96.49%, with 110 out of 114 true positives correctly identified and confidence intervals ranging from 91.26% to 99.04%. Overall, the assays show excellent performance in accurately detecting the targeted enzymes. While NG-Test CARBA 5 missed 4/21 non-carbapenemase KPC variants (namely KPC-31; -33; 66, -130), KINVO failed repeatedly to detect 3/19 IMP-variants (IMP-14, IMP-71, IMP-18, and 1/24 OXA-48-like (OXA-505) (Table 1, Figure S1B). Interestingly IMP-13 and its point mutant derivative IMP-37 were well detected using the KINVO assay, and IMP-16 was well detected using NG-Test CARBA 5, unlike previously published findings [21]. The precise identification of IMP-type metallo-β-lactamases is critical in endemic regions such as Southeast Asia, Australia, Taiwan, and Japan, where these enzymes are more frequently encountered in Enterobacterales, but also globally in non-fermenters—such as *Pseudomonas aeruginosa* and *Acinetobacter baumannii*, where IMP enzymes are notably more prevalent and their presence can significantly impact therapeutic options [13,25]. Both assays detected OXA-48 variants, displaying mutations in the ß5-ß6 loop [26,27,28], including OXA-163 and OXA-405, which are considered non-carbapenemase OXA-48 variants, but in bacteria with impaired outer membranes may lead to carbapenem-resistance [28,29].

Overall, both tests demonstrated features such as rapidity and simplicity; however, NG-Test CARBA 5 showed superior performance, particularly in accurately detecting IMP carbapenemases and the OXA-48 variant OXA-505, compared to the KINVO test. The migration was faster (especially for VIM and OXA-48-likes) with stronger band-intensities with the KINVO assay as compared to NG-Test CARBA 5, which could be related to the number of bacteria used in both assays (1–3 colonies as compared to three 1 µL loops full of bacteria for NG-Test CARBA 5 and KINVO, respectively). Unlike other LFIA tests [3,8,9] that require two or more different detection cassettes, NG-Test CARBA 5 and KINVO utilize only a single cassette, which is particularly advantageous in an environmentally conscious world. Of note is that fact that, with some isolates (nearly 10%), the migration on KINVO strips resulted in dark-red-stained nitrocellulose membranes, and even sometimes in an additional band appearing at the very lower part of the reading window, which does not align with a specific enzyme line (Figure S1C).

### 3.2. Susceptibility Testing, Carbapenem Hydrolysis Test, and KPC-Specific PCR

A previous study that used Carba NP test to monitor carbapenem-hydrolytic activity of OXA-48 variants revealed that OXA-163 and OXA-405, which have a four AA deletions in their ß5–ß6 loops, are no longer carbapenemases, but rather OXA-ESBLs [16,28]. Similarly, mutations in KPC, such as D179Y in the Ω loop, have been shown to result in loss of carbapenem-hydrolyzing activity and gain of ceftazidime hydrolysis, resulting in the bacteria expressing these enzymes having reduced susceptibility/resistance to CAZ/AVI [24]. These isolates display negative Carba NP results but are still detectable by PCR. The ability of the different KPC variants isolated at the French National Reference center between January 2019 and June 2025 (n = 519) to hydrolyze carbapenems and to confer resistance to carbapenems and/or CAZ/AVI was evaluated (Table 2). KPC is still relatively rare in France as compared to OXA-48 and NDM variants and most important cases from countries with prevalences [30]. KPC-2/KPC-3 represent nearly 98% of KPC variants isolated in France. To extend the list of KPC variants, several variants cloned in pTOPO and expressed in *E. coli* TOP10 as previously described have also been included [24]. Out of the 19 KPC variants tested, 9 lost carbapenem hydrolysis ability as revealed by negative Carba NP tests. Of these nine, all the Carba NP-negative isolates were detected by PCR, as PCR detects a gene irrespective of the associated phenotype. Similarly, the KINVO test detected all of them as KPC-type carbapenemase producers, while only five were detected by NG-Test CARBA 5. The four non-detected KPC variants by the NG-Test CARBA 5 were all susceptible to carbapenems and resistant to CAZ-AVI.

### 3.3. LFIA for Carbapenemase Detection

If one considers detection of enzymes of the big five carbapenemases displaying carbapenemase activity, the specificity would be 72% and 56% and sensitivity 100% and 96.12% for NG-Test CARBA 5 and KINVO, respectively (Table 2). Indeed, both assays mis-identify OXA-163 and OXA-405-producers as CPEs. In addition, KINVO (KPC-14, -28, -31, -33, -39, -66, -89, -130, -148) and NG-Test CARBA 5 (KPC-14; -28; -39; -89; -148) detect ESBL-type KPC variants as carbapenemases (Table 2 and Table 3).

## 4. Discussion

Enhanced surveillance, antimicrobial stewardship programs, and strict adherence to infection prevention protocols are vital components in combating the spread of CP GNB, ultimately reducing morbidity, mortality, and the burden on healthcare systems, but this requires rapid and reliable detection of these bacteria [1,2]. LFIAs are versatile and valuable diagnostic tools that offer significant clinical benefits for antimicrobial resistance (AMR) detection [5]. Their ease of use—requiring no specialized training—combined with rapid results in less than 15 min makes them highly practical in various settings. Additionally, LFIAs demonstrate high reliability, with near 100% sensitivity and specificity, especially when confirming the presence of the five main carbapenemases in cultured Gram-negative bacteria. Importantly, LFIAs can also be applied directly to clinical samples, facilitating timely and accurate detection of resistant pathogens, which is critical for effective infection control and appropriate antimicrobial therapy [5,7,31].

The emergence and spread of novel enzyme variants, primarily driven by antibiotic selection pressure, pose significant challenges in managing resistant bacterial infections [14]. In 2015, a significant advancement was made with the approval of ceftazidime (CAZ) combined with avibactam (AVI) in the United States. Avibactam is a non-β-lactam β-lactamase inhibitor that enhances the efficacy of ceftazidime against certain resistant bacteria. This combination was specifically approved for treating infections caused by carbapenemase-producing Enterobacterales, notably those producing KPC enzymes, which are often resistant to multiple antibiotics. The CAZ/AVI combination has since become a first-line treatment option for MDR bacterial infections and is recommended by several infectious disease societies for infections caused by bacteria harboring KPC and OXA-48 carbapenemases [32,33].

Along with CAZ-AVI clinical use, resistance has rapidly emerged, with mutations in *bla*KPCs being major players in CAZ-AVI resistance, alongside mutations in *bla*SHV, *bla*CTX-M, and *bla*OXA resistance genes [24,26,27,28,29,30]. Mutations occurring in the Ω-loop of KPC-2 and KPC-3 enzymes, as well as in the 270 loop, have been associated with increased resistance to CAZ-AVI [34]. Specifically, substitutions within positions 164–179 of the Ω-loop enhance enzymes’ affinity for ceftazidime, which in turn diminishes the effectiveness of avibactam by preventing its binding [24,35]. These alterations facilitate the hydrolysis of ceftazidime despite the presence of a β-lactamase inhibitor, contributing to the development of resistance [24,35].

Interestingly, KPC variants that confer CAZ/AVI resistance have lost their carbapenemase-hydrolyzing property, turning these enzymes into ESBLs, and infections with Enterobacterales producing these variants could be effectively treated with carbapenems [33].

Rapid diagnostic tests, including LFIAs, are used now to identify carbapenemases and allow early initiation of effective therapy with bloodstream infections [8,9,32]. However, detection of CAZ/AVI-resistant variants, such as KPC-31 and -33, may lead to therapeutic failure with CAZ/AVI and possible selection of resistance in other microbial species of the gut microbiota; while these isolates are no longer carbapenemases (being fully susceptible to carbapenems), they could theoretically be treated with carbapenems [33].

This study has some limitations, as even though 114 well-characterized GNB isolates including 8 VIM, 24 OXA-48, 21 KPC, 15 NDM, and 19 IMP variants were investigated, these numbers are relatively low as compared to the number of variants described in the BLDB (www.bldb.eu; >70 OXA-48, 281 KPC, 95 VIM, 111 IMP, 91 NDM variants). Evaluation on larger panels of isolates including very rare variants would increase the overall significance of the results. The isolates and the variants that have been selected in this study represent the main carbapenemases isolated in France, and likely globally, as nearly 50% of them were imported cases coming from all over the world.

## 5. Conclusions

Both LFIAs provide rapid, reliable detection of the major carbapenemases in Gram-negative bacteria, facilitating timely infection control measures and guiding antimicrobial therapy. The choice between the two may depend on regional availability, cost considerations, and specific performance nuances observed in local bacterial populations. While NG-Test CARBA 5 exhibited better performances as compared with the KINVO test, for the accurate detection of IMP and OXA-48 variants (OXA-505), the KINVO assay detected more KPC variants, including those that have lost carbapenem hydrolysis and gained ceftazidime/avibactam resistance. As these KPC variants (such as KPC-14, -28, -31, and -33) cannot be considered carbapenemases anymore, it becomes questionable whether they should be detected. As PCR and the KINVO assay detect all KPC variants irrespective of the associated phenotype, a positive test result may lead to the use of CAZ/AVI on CAZ/AVI-resistant isolates.

CAZ/AVI is recommended as a first-line treatment of KPC producers, but with the spread of variants conferring CAZ-AVI resistance and carbapenem susceptibility to Enterobacterales, KPC identification becomes challenging. Accurate identification of these variants is important, as infections with Enterobacterales expressing them may not be treated effectively with CAZ/AVI but with carbapenem-based regimens [33,35]. Combined use of carbapenemase-detection assays (biochemical assays, LFIAs, or molecular assays) is needed to detect these KPC variants, which are increasingly being detected, especially in countries with high KPC prevalence and high CAZ/AVI consumption to treat infections with KPC-producers.

## Figures and Tables

**Table 1:** LFIA test results.

Mechanisms of Resistance	Species	NG-Test Carba5 (NG Biotech)		KINVO (Medomics Medical Technology)	
**N isolates = 114**		**Result**	**Time (Intensity)** **^a^**	**Result**	**Time (Intensity)** **^a^**
Non-carbapenemase-producers (n = 5) Case + impermeability (2); plasmid-encoded Case (1); CTX-M + impermeability (2)	*E. coli* (2); *K. pneumoniae* (2); *E. cloacae* (1)	Negative ^b^	3′40′′ (+/−6′′) for control line ^b^	Negative	1′40′′(+/−12′′) for control line ^b^
Rare carbapenemases (n = 9); SME-2, IMI-1, GES-5, FRI-1, OXA-23, OXA-372, LMB-1, TMB-1, GIM-1	*E. cloacae* (5); *C. freundii* (2); *S. marcescens* (1); *P. mirabilis* (1)	Negative	3′20′′ (+/−35′′) for control line ^b^	Negative	1′40′′(+/−9′′) for control line ^b^
KPC-type (n = 21); KPC-2 (2), -3 (2), -4, -5, -6, -12, -13, -14, -15, -23 (2), -28, -31, -33, -39, -66, -89, -130, -148	*E. coli* (11); *K. pneumoniae* (9); *S. marcescens* (1)	17/21 (81%); not detected: KPC-31; -33, -66, -130 ^c^	2′46′′ (+/−60′′) (G7–G8)	21/21 (100%)	2′10′′ (+/−80′′) (G5–G7)
NDM-type (n = 15); NDM-1 (2), -2 (2),-4, -5, -6, -7, -9, -14, -19, -24, -29, -35, -39	*E. coli* (10); *K. pneumoniae* (2); *A. baumannii (3)*	15/15 (100%)	3′06′′ (+/−140′′) (G7–G8)	15/15 (100%)	2′50′′ (+/−140′′) (G8)
VIM-type (n = 8); VIM-1 (2), -2 (2), -4 (2), -5, -19	*E. coli* (2); *K. pneumoniae* (2); *P. aeruginosa* (2); *E. cloacae* (2)	8/8 (100%)	3′6′′ (+/−140′′) (G7–G9)	8/8 (100%)	28′′ (G10)
IMP-type (n = 19); IMP-1 (3), -2, -7, -11, -13, -14, -15, -16, -19, -26, -29, -31, -37, -58, -63, -71, -94	*E. coli* (1); *K. pneumoniae* (1); *P. aeruginosa* (12); *Acinetobacter* spp. (3); *C. freundii* (1); *S. marcescens* (1)	19/19 (100%)	1′50′′ (+/−72′′) (G8–G9)	17/19 (89%); not detected: IMP-14; IMP-71	1′33′′ (+/−88′′) (G9–G10)
OXA-48-like (n = 24); OXA-48 (3), 162, -163, 181 (2), 204 (3), -232 (2), -244 (3), -370, -405; -484, -515, -517, -519, -535, -793, -933	*E. coli* (9); *K. pneumoniae* (10); *C. freundii* (2); *S marcescens* (1); *Shewanella sp*. (2)	24/24 (100%)	1′53′′ (+/−60′′) (G8–G9)	24/24 (100%) (G9–10)	34′′ (G9–G10)
Multiple carbapenemases (n = 13); NDM-1 + OXA-48 (2) NDM-1 + OXA-232 NDM-5 + OXA-232 NDM-5 + OXA-181 NDM-1 + VIM-2 OXA-48 + VIM-4 NDM-7 + KPC-4 NDM-4 + KPC-2 OXA-48 + KPC-28 OXA-181 + NDM-5 + VIM-1 OXA-505 + VIM-1 IMP-18 + VIM-2	*E. coli* (4); *K. pneumoniae* (5); *C. freundii* (1); *E. cloacae* (2); *P. aeruginosa* (1);	13/13 (100%)	For NDM (1′15′′)(G8) KPC (3′08′′) (G7–8) OXA48-like (2′02′′) (G9), IMP (27′′) (G9) and for VIM (1′56′′) (G8–G9)	11/13 (85%); not detected: IMP-18; OXA-505	For NDM (1′33′′) (G8) KPC (1′55′′) (G9) For OXA48-like (21′′) (G10), and for VIM (1′03) (G9)

^a^: Average time (min) to visible band, and intensity of bands at 15 min reading according to intensity ruler from NG-Biotech (see Figure S1B), going from G1 (hardly visible by eye) to G10 (very intense). ^b^: Negative test result as observed after 15 min. ^c^: Not-detected variants.

**Table 2:** Test performances.

	NG-Test Carba5 (NG Biotech)		KINVO (Medomics Medical Technology)	
	Big 5 Variant Detection ^a^	Big 5 Variant Detection with Carbapenemase Activity ^b^	Big 5 Variant Detection ^a^	Big 5 Variant Detection with Carbapenemase Activity ^b^
Specificity	100% (CI = 76.84–100%)	72.00% (CI = 50.61–87.93%) Non-carbapenemases detected (7): KPC-14, -28, -39, -89, -148, and OXA-405, -163	100% (CI = 76.84–100%)	56.00% (CI = 34.93–75.60%) Non-carbapenemases detected (11): KPC-14, -28, -31, -33, -39, -66, -86, -89, -148, and OXA-405, -163
Sensitivity	96.49% (110/114, CI = 91.26–99.04%) Not detected: KPC-31; -33, -66, -130	100% (103/103, CI = 96.70–100%)	96.49% (110/114, CI = 91.26–99.04%) Not detected: IMP-14 IMP-71 IMP-18 OXA-505	96.12% (99/103, CI = 90.35–98.93%) Not detected: IMP-14 IMP-71 IMP-18 OXA-505
Younden’s Index ^c^	96.49	72.00	96.49	49.12

^a^ The sensitivities, and specificities were calculated with their respective 95% confidence intervals (95%CI) using the free web-based software MedCalc (https://www.medcalc.org/calc/diagnostic_test.php, accessed on 25 July 2025). The Gold Standard was WGS data. These values were calculated for detection of the 114 KPC, VIM, IMP, OXA-48, and NDM variants present. ^b^ Test performances were calculated for the 103 KPC, VIM, IMP, OXA-48, and NDM variants with significant carbapenemase activity. OXA-405 and -163 and KPC-14, -28, -31, -33, -39, -66, -89, -130, and -148 are considered expanded-spectrum hydrolysing enzymes and not carbapenemases [16,24]. ^c^ Youden’s index (sensitivity (%) + specificity (%) − 100) calculates the efficiency of a diagnostic test; a Youden score of 100 indicates a perfect test.

**Table 3:** KPC variants included in this study.

				Antimicrobial Agents ^c^												Confirmation Assays			
	KPC Variants	AA Changes ^a^	KPCs at F-NRC ^b^ (2019–June 2025; n = 519)	AMX	AMC	CRO	CTX	CAZ	CAZ/AVI	CFX	FEP	ATM	IMP	MEM	ERT	Carba NP Test	*bla*_KPC_ PCR	NG-Test CARBA 5	KINVO
*E. coli* TOP10 pTOPO-	KPC-2		147 (27.80%)	>256	24	12	8	4	0.38	3	2	24	8	3	1	+ ^f^	+	+	+
	KPC-3	H273Y	360 (69.80%)	>256	48	48	>32	>256	0.75	12	6	>256	8	3	1	+	+	+	+
	KPC-4	P104R, V240G	2 (0.4%)	>256	nd	>256	8	>256	0.5	>256	2	>256	1	0.25	0.19	+	+	+	+
	KPC-5	P104R	0	>256	nd	>256	8	>256	0.5	>256	2	>256	1	0.25	0.19	+	+	+	+
	KPC-6	V240G	0	>256	nd	48	>32	32	0.75	16	4	>256	4	3	1	+	+	+	+
	KPC-12	L169M	0	>256	nd	6	4	4	0.38	3	1	12	0.75	0.25	0.125	+	+	+	+
	KPC-13	D92G, H273Y	0	>256	nd	12	4	4	0.25	4	2	64	1.5	0.5	0.38	+	+	+	+
	KPC-14	ΔGT242–243	0	>256	6	6	6	>256	24	12	4	32	0.25	0.032	0.006	−	+	+	+
	KPC-15	A120L, G147K	0	>256	nd	16	6	3	0.38	3	2	32	6	1.5	1.5	+	+	+	+
	KPC-23	V240A, H273Y	1 (0.2%)	>256	nd	64	>32	12	0.38	12	4	>256	8	2	1.5	+	+	+	+
	KPC-28	H273Y, ΔGT242–243	0	>256	6	4	4	>256	12	8	4	24	0.25	0.032	0.008	−	+	+	+
	KPC-31	H273Y, D179Y	1 (0.2%)	6	nd	2	1.5	>256	12	4	1	0.75	0.25	0.032	0.008	−	+	−	+
	KPC-33	D179Y	0	12	nd	1	1.5	>256	8	6	1	0.75	0.25	0.032	0.008	−	+	−	+
	KPC-39	A171T, H273Y	1 (0.2%)	>256	24	2	2	>256	4	4	2	>256	1	0.094	0.094	−	+	+	+
*K. pneumoniae* 333D2	KPC-23 (OXA-9, SHV-11, TEM-1)	V240A, H273Y	1 (0.2%)	>256	>256	6	32	48	0.5	nd ^d^	8	64	4	0.5		+	+	+	+
*K. pneumoniae * 347G8	KPC-66 (CTX-M-15, OXA-1, SHV-28, TEM-1)	ΔEL168–169	1 (0.2%)	>256	>256	>32	>32	>256	8	nd	>256	>256	0.75	6 ^d^	>32 ^e^	−	+	−	weak
*E. coli * 375G3	KPC-130 (ESC-312, TEM-135)	D179G, H273Y	3 (0.6%)	>256	>256	>32	>32	>256	>256	nd	16	8	1	0.094	0.19	−	+	−	+
*K. pneumonie * 431C6	KPC-89 (SHV-11)	T243M	1 (0.2%)	>256	>256	12	>32	>256	16	nd	48	48	4	16	>32	−	+	+	+
*K. pneumoniae* 493A4	KPC148 (SHV-11)	InsS182; Ins268–269 KDDKHSEAVYTRAPN, H273Y	1 (0.2%)	>256	>256	12	6	>256	>256	nd	8	1	0.125	0.125	0.25	−	+	+	+
*E. coli* TOP10				6	6	0.032	0.064	0.125	0.125	0.38	0.064	0.047	0.25	0.032	0.004	−	−	−	−
EUCAST breakpoints 2025	S ≤; R > (mg/L)			8; 8	8; 8	1; 2	1; 2	1; 4	8; 8	1; 1	1; 4	1; 4	2; 4	1; 8	0.5; 0.5				

^a^ Numbering according to Ambler alignment (Figure 1). ^b^ Number of KPC-producing isolates in the French F-NRC (2019-juin2025) n = 519. ^c^ AMX: Amoxycillin; AMC: Amoxy-clav; CRO: ceftriaxone; CTX; cefotazime; CAZ: ceftazidime; CAZ/AVI: ceftazidime/avibactam; CFX: cefixime; FEP: cefepime; ATM: aztreonam; IMP: imipenem; MER: meropenem; ERT: Ertapenem. MICs were determined using E-Tests (BioMérieux) and interpreted according to EUCAST breakpoints 2025 guidelines. Dark grey colored cells correspond to Resistance, light grey to intermediate, and white to susceptible For AMX and AMC IV breakpoints, and for CFX, noncomplicated urinary tract infection breakpoints were used. ^d^ nd: Not done. ^e^ Heteroresistance, ellipse with growing colonies. ^f^ ‘+’ stands for positive test result, and ‘−’ for a negative test result.

## Data Availability

The original contributions presented in this study are included in the article/Supplementary Material. Further inquiries can be directed to the corresponding author.

## Supplementary Materials

The following supporting information can be downloaded at: https://www.mdpi.com/article/10.3390/microorganisms13092140/s1, Figure S1: LFIA test results. Panel A: KINVO Test from Medomics (1), and NG-TEST CARBA 5 from NG-Biotech (2); Panel B: Discordant results using KINVO Test and NG-TEST Carba5 for *K. pneumoniae* KPC-66; *K. pneumoniae* KPC-33; *P. aeruginosa* IMP-18 + VIM-2; *P. aeruginosa* IMP-94; and *C. freundii* VIM-1 + OXA-505. V, I, N, O, K, and C stand for VIM, IMP, NDM, OXA-48, KPC and Control test line, respectively. Pictures were taken after 15 min migration. Panel C: Adverse events observed for some isolates with KINVO strips. Dark red stained membrane and additional band at lower part of reading window; Panel D: Intensity ruler from NG-Biotech. After 15 min migration, the intensity of each band has been compared by eye to those of the intensity ruler, and scored accordingly (see Table 1).
