# Supplementary material for: Comparison of Two Lateral Flow Immunochromatographic Assays for Rapid Detection of KPC, NDM, IMP, VIM and OXA-48 Carbapenemases in Gram-Negatives
Source: Microorganisms. 2025 Sep 12;13(9):2140. doi: 10.3390/microorganisms13092140 (PMC12472453; doi:10.3390/microorganisms13092140)
Supplement: Supplementary file 1 [file microorganisms-13-02140-s001.zip › 2-Supplemental figure S1.pdf]

Supplemental figure S1

Comparison of two lateral flow immunochromatographic assay for the rapid detection of KPC, NDM, IMP, VIM and OXA-48 carbapenemases in Gram-negatives

Clara MORALES DOMINGUEZ,[1,2,3] Saoussen OUESLATI,[4,5] Nahed AL LAHAM,[4,6] Réva NERMONT,[4] Hervé VOLLAND,[7] Thierry NAAS[4,5,8*]

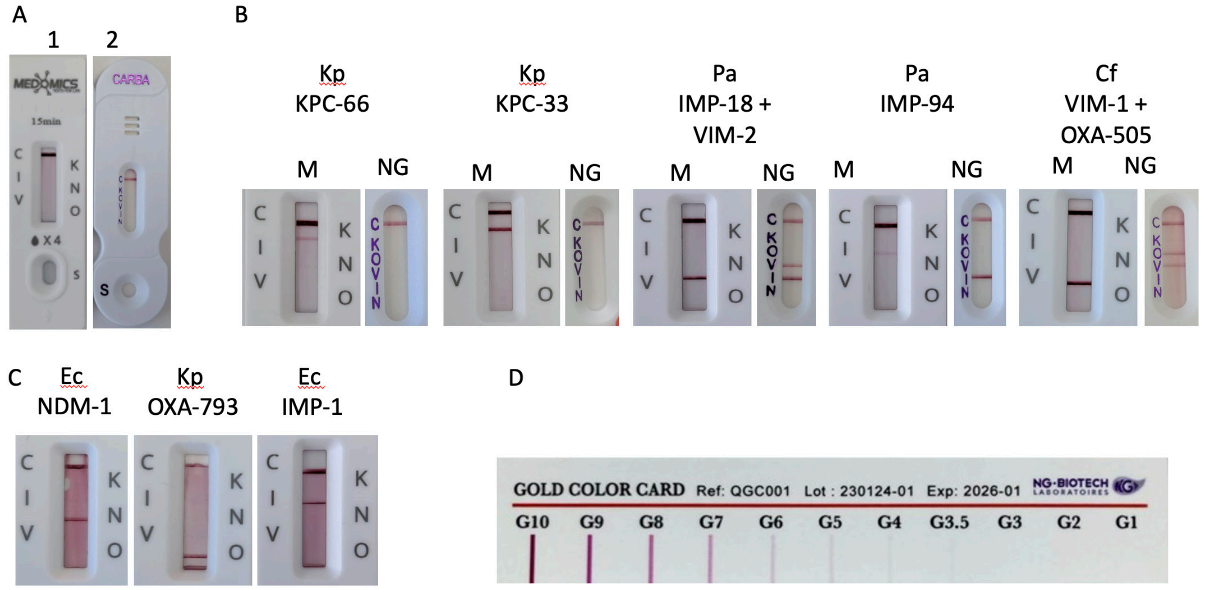

Figure S1: LFIA test results. Panel A, KINVO Test from Medomics (1), and NG-TEST CARBA 5 from NG-Biotech (2); **Panel B**: Discordant results using KINVO Test and NG-TEST Carba5 for *K. pneumoniae* KPC-66; *K. pneumoniae* KPC-33; *P. aeruginosa* IMP-18 + VIM-2; *P. aeruginosa* IMP-94; and *C. freundii* VIM-1 + OXA-505. V, I, N, O, K, and C stand for VIM, IMP, NDM, OXA-48, KPC and Control test line, respectively. Pictures were taken after 15 minutes migration. Panel C: Adverse events observed for some isolates with KINVO strips. Dark red stained membrane and additional band at lower part of reading window; Panel D: Intensity ruler from NG-Biotech. After 15 minutes migration, the intensity of each band has been compared by eye to those of the intensity ruler, and scored accordingly (see Table 1);
